# Innovations in geomatics teaching during the COVID-19 emergency

**DOI:** 10.1007/s12518-022-00416-4

**Published:** 2022-01-17

**Authors:** Matteo Botto, Bianca Federici, Ilaria Ferrando, Sara Gagliolo, Domenico Sguerso

**Affiliations:** 1 Department of Educational Sciences (DISFOR), University of Genoa, Genoa, Italy; 2 Geomatics Laboratory, Department of Civil, Chemical and Environmental Engineering (DICCA), University of Genoa, Genoa, Italy

**Keywords:** Geomatics teaching, Educational innovation, Student involvement, e-Learning, Moodle, Distant learning, Remote teaching, Team-based learning, Problem-based learning

## Abstract

The approach in the teaching process is changing, thanks to the increased awareness that a higher students’ involvement leads to a better quality of their learning. The aim is to make the students more participative, avoiding a unidirectional lesson and encouraging their wish to keep updated on the course advancements. However, innovative teaching methodologies are not yet widespread, mainly in STEM (Science, Technology, Engineering, and Mathematics) disciplines. At the University of Genoa, the experimentation of innovative teaching techniques has been significant and worthy especially because it was planned before the COVID-19 emergency and applied in the scenario of forced remote teaching. Thanks to the introduction of novel technological instruments, several techniques have been exploited to realize interactive lessons and promoting students’ involvement. The present work discloses the employed techniques and frames them within the state of the art of innovative teaching, highlighting their contribution in the teaching activities related to the Geomatics field of knowledge. The acquired experiences in Geomatics dissemination and a critical analysis, including teachers’ and students’ perception about the tested innovative teaching/learning tools, are also reported. In general, the innovations introduced in teaching and learning processes during the COVID-19 sanitary emergency were warmly received by the entire community, including teachers, students, and teaching assistants.

## Introduction

As widely known, Geomatics is the discipline that deals with survey, monitoring, and analysis of the natural and built environments from the metric and thematic points of view. Geomatics aims to provide a correct knowledge of the territory and environment; therefore, it is required in many circumstances: various surveying techniques and methodologies can be applied for detailed surveying of the position of objects, like technological networks or vehicles (Marzocchi et al. 2017; Cristodaro et al. 2019; Gonzalez and Dabove 2019; Leppälä et al. 2019); for the production of cartography of territorially extended areas; for the monitoring of structures, infrastructures, and/or the natural environment (Bovolenta et al. 2017; Minghini et al. 2017; Gagliolo et al. 2018; Themistocleus and Danezis 2018; Gagliolo et al. 2019; Gobbi et al. 2019; Barrile et al. 2020; Campos et al. 2021; Ferrando et al. 2021; Vaccaro et al. 2021); but also for innovative applications (Ferrando et al. 2018; Dabove et al. 2019; Piazza et al. 2019; Di Pietra et al. 2020; Rossi et al. 2020; Benvenuto et al. 2021). The instruments vary from traditional topographic methodologies to GNSS (Global Navigation Satellite System) positioning and from photogrammetry, also using UAV (unmanned aerial vehicle), to laser scanner technology. The analysis of spatially distributed data, acquired by sensors on the Earth or thanks to the elaboration of signals and images coming from satellites, could be performed in GIS (geographic information system). In addition, Geomatics uses statistical tools for the critical analysis of the measurement campaigns or observed data, to achieve reliable and verifiable results, also by integrating different data sources, if possible and if necessary, according to the relevance of the survey. Geomatic education deals with an entire production process, from design and execution to elaboration and representation. The direct contact with the operational reality, the methodological approach, and the importance given to the quality analysis of the results make the geomatics approach useful also in different fields of knowledge.

In recent years, increasing interest has been addressed to the field of innovative teaching techniques, to make the teaching process more effective and attractive and more student-centered. Within the Laboratory of Geodesy, Geomatics and GIS (hereafter, Geomatics Lab) of the University of Genoa, this issue has been approached quite in advance, as described in the “Geomatics teaching and dissemination” section, in relation to the so-called “Third Mission” (*Terza Missione*^1^) of the University, for the diffusion and dissemination of knowledge in the society at different levels. In fact, what emerged from the “Third Mission” experiences was fundamental in extending the innovative educational approach even in the academic scenario, as described in the following. Also, innovative teaching is important to involve all the possible learning styles of the students (Kolb and Kolb 2005) and to contribute to the achievement of the Sustainable Development Goal n. 4 (Ensure inclusive and equitable quality education and promote lifelong learning opportunities for all) of the 2030 United Nations agenda.

At the beginning of the academic year 2019/2020, the Department of Civil, Chemical and Environmental Engineering (DICCA) of the University of Genoa has proposed to introduce innovative aspects of teaching, independently and in advance to the COVID-19 health emergency. This decision was coherent with the ongoing didactic innovation in Europe (noteworthy examples in Geomatics area are Kosmatin Fras and Grigillo 2016; Martín-Romero et al. 2018; Tucci et al. 2019) and to the wish of a more effective feedback and check about the understanding achieved by the students. The COVID-19 outbreak speeded up the ongoing evolution by requiring some changes to the pre-established schedule.

In Geomatics teaching, the experimentation involved both basic courses on Geomatics and GIS, and more specialized ones, dedicated, for example, to Monitoring techniques or to Photogrammetry and Remote Sensing. In order to introduce innovative tools in an incisive way, a collaboration was established between the Geomatics Lab and a high-level expert in teaching aspects. The initial need to describe the Geomatics peculiarities to this professional figure, external to the Geomatics subjects, allowed to better define the training objectives of the courses. Subsequently, salient points were identified on which to focus the attention, mainly aiming to make the students understand the primary concepts of their training.

The present work is intended to describe new teaching strategies deployed in Geomatics disciplines, highlighting which novelties were already planned and what has been rapidly conceived and implemented adapting to the remote online teaching imposed by the COVID-19 outbreak. In the “Geomatics teaching and dissemination” section, a hint on previous experiences of our team in Geomatics teaching and dissemination is reported together with an overview on the innovative teaching techniques already applied in Geomatics teaching. The didactic strategies and tools applied in the 2019/2020 academic year are described in the “Adopted instruments and their relationship with the course’s peculiarities” section. A critical analysis coming from teachers’ and students’ perception about the experimented didactic tools is presented in the “Teachers’ and students’ perception on the experimented innovative didactic tools” Sect. 4. Concluding remarks to our didactic experience are reported in the “Conclusions” section.

## Geomatics teaching and dissemination

This section proposes an overview of innovative teaching experiences in Geomatics (“Innovative didactics in Geomatics disciplines” section) and on the previous experiences carried out by the Geomatics Laboratory of Genoa University before the COVID-19 sanitary emergency that required forced distance learning (“Previous dissemination experiences of the Geomatics Lab” and “A virtuous cycle: practical activities for didactics and didactics as inspirations for future research and work” sections).

### Innovative didactics in Geomatics disciplines

Innovative didactic techniques have already been tested and applied on STEM (Science, Technology, Engineering, and Mathematics) disciplines and on Geomatics teaching. The several experimented techniques, ranging from PBL (problem-based learning) and PBeL (problem-based e-learning) to CBL (challenge-based learning), from FC (flipped classroom) to LBD (learning by doing), have the common final aim of easing the learning process, mainly in relation to the more difficult theoretical concepts, and to make students more and more interested and passionate in the Geomatics disciplines. Indeed, on the one hand, the overall public is gaining interest on Geomatics, mainly in the activities involving the survey, i.e., laser scanning, photogrammetry, and GNSS, also thanks to the spreading of mass-market technological instrumentation, and, on the other hand, it seems necessary to attract students in pursuing STEM study courses. As a matter of facts, the innovative didactic techniques could play a key role in catching the young generations’ attention, proposing a non-standard way of learning and stimulating them to enroll in science degree courses, that suffer from a lower number of students in the last years with respect to human sciences study courses (Becker 2010).

In the recent years, several virtuous examples to introduce the main concepts of Geomatics disciplines have been successfully carried out, which addressed to students (Gil-Docampo et al. 2019; Tucci et al. 2019), technicians (Tucci et al. 2018), and non-specialist public (Bonacini et al. 2015; Ortiz-Sanz et al. 2021). In this context, mainly photogrammetry has gained more and more popularity, also thanks to the spread of “toy drones” and automatic photogrammetric software (desktop, mobile app, and online), that allow the users to obtain realistic and pleasant 3D models without the necessity of knowing the basic concepts of photogrammetry but only taking pictures in the appropriate way, at least paying attention to the overlapping of images and the light conditions (Tucci et al. 2020). This produces two effects: (1) people become more and more interested and involved in these topics because of the spreading of low-cost hardware and software solutions for managing the entire photogrammetric process and its perceived easiness, from scene capturing to 3D model reconstruction; (2) the theoretical concepts remain unknown and are rarely deepened, producing an increasing gap between theory and application of photogrammetry and, in the worst cases, a trivialization of the entire photogrammetric process. Moreover, the bigger criticality from the geomatic point of view is the lack of parameters associated to the final model to describe its quality, demonstrating the lack of awareness of need to control the quality of the outputs.

The main resistance in studying and deepening Geomatics concepts comes from the perceived hostility and complexity of the topics, which are often considered too theoretical or specialized. In this sense, innovative didactic methods, concerning both teaching–learning processes (König et al. 2012; Tucci et al. 2020) and introduction of technological devices (Mills 2015; Luhmann 2016; Ortiz-Sanz et al. 2021) are beneficial in making people approach geomatics topics in a smoother and more engaging way and in moving the role of the teacher to facilitator of the learning process, reducing the gap between teacher and students. Moreover, the learning strategies that promote collaboration and direct interaction between students have the further advantage of helping them in building and strengthen their soft skills: teamwork, communication, problem-solving, creativity.

The proposed innovative teaching–learning methodologies resulted extremely useful also during the COVID-19 sanitary emergency (Hebebci et al. 2020), allowing a more globalized and flexible access to knowledge, without limits of time (the lessons are typically registered, so that a student can watch them anytime) and space (the lessons can be followed in any place, without being necessarily in the classroom, provided that the internet connection is available). The introduction of the innovative methodologies before the forced distant teaching and learning due to COVID-19 outbreak was particularly fruitful because the students and the teachers were already prepared to the new approach and quite used to employ the technological instruments. This is even more favorable in sight of the new trend of blended learning that couples the traditional frontal lesson setting and the use of e-learning contents, i.e., interactive textbooks, videos, webinars, and online tutorials, to improve the teaching effectiveness and to increase the students’ engagement, also with the advantage of a smarter and more flexible accessibility and smoother way of updating the courses materials with respect to traditional printed textbooks and lecture notes.

It must be noted that the innovation in Geomatics teaching, and in all the STEM disciplines, is an ongoing process. It is evident that it is not only related to the introduction of technological devices, e.g., computers, tablets, and mobile phones, just to access the lessons materials or to take notes, but it involves the entire teaching–learning paradigm in a more appealing, engaging, and effective way. In this context, the technological devices act as didactic tools that can be used in Geomatics field exploiting their embedded sensors (Tucci et al. 2020) and transforming the mobile device in a measurement tool (Ortiz-Sanz et al. 2020) that can help the teachers in finding new strategies for transmitting the fundamental concepts of geomatics disciplines.

### Previous dissemination experiences of the Geomatics Lab

The experimentation of new educational strategies by the Laboratory of Geomatics of Genoa University started with several workshop offered to different audiences, mainly dedicated to the altitude measurement and to the photogrammetric technique. They were proposed during didactical events for kids (*UniversiKids*^2^), cultural festivals for students from primary to high schools (*Festival del Mare*^3^), and for general public (*Notte Bianca di Savona*), in addition to seminars for citizens (*Pint of Science*,^4^*Mercoledì Scienza by Amici dell’Acquario*^5^), for university students, for senior (“Third Age”) university students, and for professionals (Sguerso et al. 2020). The educational activities contemplated a theoretical part, where the objectives and motivations were introduced by using posters, videos, and explanations to clarify the main concepts. A more practical part followed, including short experiments and experiences accompanied by a full scientific explanation. Therefore, it could be assimilated to the teaching strategy of problem-based learning (PBL), as described in the “PBL—problem-based learning” section.

Such experiences strongly showed the audience’s wish to be an active part of the learning process, as well as the necessity to focus on the public needs using easily understandable language, practical examples, clear explications, closeness to social needs, and high emotional involvement. Moreover, the contribution of high school students in internship at the University (the so-called *Alternanza Scuola-Lavoro*,^6^*AS-L*, literally “*School-Work Alternation*”, evolved on 4^th^ September 2019 in *Percorsi per le Competenze Trasversali e per l’Orientamento,*^7^* PCTO*, literally “*Paths for Transversal Skills and Orientation*”), who were involved in the preparation and management of the events, was highly constructive and productive. They felt as an active part of the learning process, relying both on their previous knowledge and critical thinking and collecting and retransmitting the learned concepts.

Therefore, it can be stated that the mentioned events have involved three different types of stakeholders. The first is the public, who is entertained and made curious to understand the basic scientific concepts, starting from intuitive considerations and questions of the audience itself. The second is represented by high school students on internships at the university and involved in the events, having the chance to improve their soft skills, such as communication, teamwork, problem-solving, and creativity. Finally, the third kind of stakeholders is represented by the members of the Geomatics Lab, who benefit from the didactic experimentation, being aware that “the best way to learn is teaching.”

**Fig. 1:**
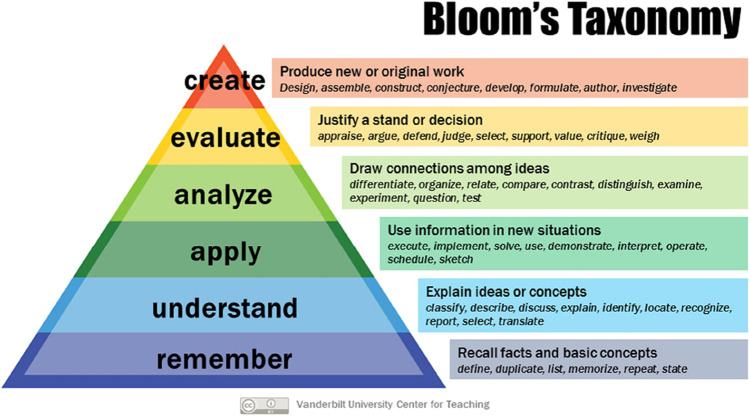
The learning pyramid by the Bloom’s Taxonomy

This last sentence is confirmed by the famous Bloom’s Taxonomy (Anderson and Krathwohl 2001). Knowledge is at the basis of these six cognitive processes, as Fig. 1 shows. By climbing the pyramid from its base, the learning method becomes more and more interactive, and students’ skills increase. The activities at the top require the students to be more active (like doing a presentation or managing work in groups) and potentially promote more significant learning. This happens because the teacher does not simply ask to “remember” something, but to “understand” it, to “apply” the concept in new context, to “analyze” and “evaluate” the results, and eventually to “create” an original work. In particular, teaching to other groups requires a thorough understanding of the subject, not only by remembering concepts, but by looking for answers to possible questions. On the contrary, the activities at the basis of the pyramid are more passive and require less involvement of the students.

### A virtuous cycle: practical activities for didactics and didactics as inspirations for future research and work

The combination of theoretical and practical approaches plays a relevant role in Geomatics courses, whether the focus is on surveying techniques or spatial data management. It involves many specific competences and, at the same time, requires a careful critical analysis of the entire process, quantifying the accuracy of each step, from the design to the 2D/3D representation of the results. Hence, practical activities, typically led in outdoor environment for the survey campaigns and in computer classroom for the processing of data and for GIS analysis, are considered particularly useful for learning the topics addressed in class, approaching their practical application, and for acquiring the necessary sensitivity.

Over the years, Geomatics teachers at the Genoa University have always been used to combine the theoretical lessons with laboratory/practical experience, paying particular attention in providing to the students such knowledge and skills suitable to face geomatics issues, in coherence with the recent research developments and achievements concerning software packages, instrumentation, and strategies.

The participation of students in a survey campaign allows them both to highlight best practices and key issues in planning operations and data collecting and to analyze the challenging features and aspects related to the proposed scenario, which is typically a real site chosen according to the specific field of interest (e.g., architectonic, structural, environmental) for the students with a higher level of preparation, i.e., Master’s Degree courses.

During the post-processing phase, accomplished in laboratory by means of specific software packages, e.g., for photogrammetric purposes (Agisoft Metashape©), for point clouds management (CloudCompare), or for GIS analysis (GRASS GIS and QGIS), the students are initially guided to learn the criteria by which they can achieve the expected result. Then the students are required to form small groups (from 2 to 4 persons) and produce a technical report on the practical activities carried out. The presentation of this report and its critical discussion during the final examination is considered preparatory to the students’ future working life, enabling them to better acquire the ability to share ideas and work as a team. Moreover, in photogrammetric experiences, where all the participants start with the same datasets, the chance of comparing the results of different groups of students, that are usually slightly different due to the personal interpretation of the guidelines provided by the teachers, let the achievement of a sort of benchmark.^8^

The students’ attendance at dedicated survey campaigns is often inspiring them to further deepen geomatics topics in their thesis, both for professional and research future perspectives. They acquire skills in managing the entire workflow, from the initial planning to the final production of the required outputs, presenting their results in a conscious way.

Furthermore, the didactic experience goes hand in hand with the opportunity for the teachers to submit to the students their research outcomes for multiple purposes, like testing and validating procedures, applying tools for specific tasks, and giving birth to a virtuous cycle in which all the working areas are compenetrating and integrated. In the specific case, the application of tools born within the Geomatics Laboratory has been proposed, like G4M (GNSS for Meteorology; Ferrando 2017) for producing 2D maps of the water vapor content in GIS environment, U.Ph.O. (Unmanned Photogrammetric Office; Passoni 2019) for unmanned aerial vehicle (UAV) mission planning specifically addressed to the estimation of the expected accuracy based on a network simulation, MAGO (Adaptive Mesh for Orthophoto Generation; Gagliolo 2021) for the obtainment of high-resolution orthophotos of adjacent walls, r.inund.fluv, plugin of GRASS GIS for flood-prone areas evaluation (Marzocchi et al. 2014), and an Integrated Hydrological Geotechnical model implemented in GRASS GIS for evaluation of areas susceptible to landslide triggered by rainfalls (Bovolenta et al. 2017). In all these cases, the students have been involved, so far individually, to take a primary role in performing validation tests and practical experimentations.

## Adopted instruments and their relationship with the course’s peculiarities

Geomatics teachers at the Genoa University have expressed interest in innovative methodologies so to involve students more closely during the courses and to allow them to verify the acquisition of the main concepts, to be better prepared for the professional life (simulated during the final examinations). Hence, in the 2019/2020 academic year, several innovative teaching tools have been identified and proposed, taking care not to weigh down the cognitive load of the students. The used tools are introduced in the following dedicated sections.

The experimentation involved the following courses, whose main features are summarized in Table 1: Geomatics, Geomatics for Monitoring, Operative GIS Tools, Numerical Cartography and GIS, and Photogrammetry and Remote Sensing. Note that both the GIS courses (“Operative GIS Tools” and “Numerical Cartography and GIS”) involve students coming from different fields and study courses, demonstrating once again the multi-disciplinarity of Geomatics and its applications.

**Table 1:** Geomatics courses involved in innovative teaching experimentation during academic year 2019/2020

Course	Type of course	Year	Study course	Number of students
Geomatics	Core	2^nd^ BSc	Civil and Environmental Engineering	60
Operative GIS Tools	Elective	3^rd^ BSc	Civil and Environmental Engineering Architectural Science Geological Science	30
Geomatics for Monitoring	Elective	2^nd^ MSc	Civil Engineering	< 5
Numerical Cartography and GIS	Core	2^nd^ MSc	Environmental Engineering Hydrography and Oceanography	60
Photogrammetry and Remote Sensing	Advanced	2^nd^ MSc	Hydrography and Oceanography	5

### Instant poll

Instant poll tools (or classroom questions) are training assessment methods to be used in classroom during the lesson, often associated with traditional training methods such as face-to-face teaching, to stimulate student motivation and involvement. These tools stimulate students to reflect on the content of the lesson without being passively subjected to it and can help in highlighting unclear aspects and improve learning. The methodology typically consists in showing a question on a just covered topic (or covered in a previous lesson) using an online polling platform that allows to show the distribution of the answers in real time. Before giving the solution, the teacher stimulates discussion with and among the students.

During the GIS courses, the instant poll was used to stimulate a discussion between teacher and students on the most important and difficult concepts. With the interactive web tool Wooclap,^9^ the teacher easily builds questions for students, using several question styles such as open questions,^10^ Word Cloud,^11^ matching,^12^ multiple choice,^13^ find a number,^14^ and sorting.^15^ Questions concerned geographical data definitions and formats, scale, and accuracy of cartography, planimetric and altimetric reference systems, most popular Datums, DTM (digital terrain model), digital image resolutions, database structure, and GeoWebServices. At the beginning of each theoretical lesson, the teacher activated the tool via web and asked students to live answer few questions related to the previous lesson. The answers were anonymous; therefore, the students cannot be evaluated. The teacher then shared and discussed with the students the given answers or their statistics, indicating which ones were correct. In this way, the students are more stimulated to follow the lessons carefully, being able to check the level of understanding and completeness of their own learning.

In Geomatics courses, the approach was slightly different. The teacher activated the specific tool Word Cloud at the end of each lesson, so to live ask to the students to cite the main concept of the lesson. The answers were used by the teacher at the beginning of the following lesson to reintroduce the topics, reorganizing the cloud of words.

**Fig. 2:**
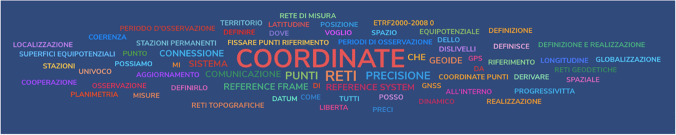
Example of a Word Cloud of a Geomatic lesson

**Fig. 3:**
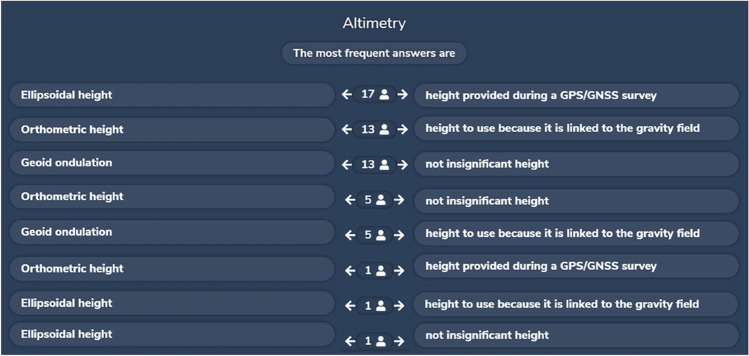
Results of an instant poll using “Matching” question style in a GIS lesson

Examples of a Word Cloud created during a Geomatic lesson and the results of an instant poll using “Matching” question style during a GIS lesson are reported in Figs. 2 and 3, respectively.

### Quiz

The quiz activity can represent a valid aid to verify the learning process, both from teacher’s and students’ sides, allowing the students’ evaluation and self-assessment (Hattie 2009). The use of quizzes is a part of the methodology called *interactive sequential instruction*, and their efficacy is based on the theory of *cognitive load* (Sweller 1988; Clark et al. 2006) that affirms the importance of basing the didactic on the level of expertise of the students, suggesting to consider the limits of the working memory to develop the opportunities of comprehension and learning.

The Quiz was firstly introduced in the Photogrammetry and Remote Sensing course, and afterwards it was employed also in the Geomatics for Monitoring course, roughly exploiting the same questions on photogrammetry topics. The quizzes were designed, built, and distributed through the Moodle^16^ course page, exploiting its capability to create quizzes comprising questions of various styles.

The criteria underlying the quiz creation can be summarized as:

- Covered topics
- Number of quizzes and questions
- Question styles (true/false, multiple choice, etc.)
- Questions weighting, maximum grade for each quiz, and marks
- Quiz accessibility and restrictions

All the criteria were explained and motivated to the students.

Four quizzes were conceived and proposed at the end of likewise didactic units. The quizzes consisted of five questions each, concerning the main themes addressed during the lessons. Several types of questions styles were proposed: multiple choice, matching, drag and drop into text,^17^ numerical questions, true/false,^18^ and missing words.^19^

**Fig. 4:**
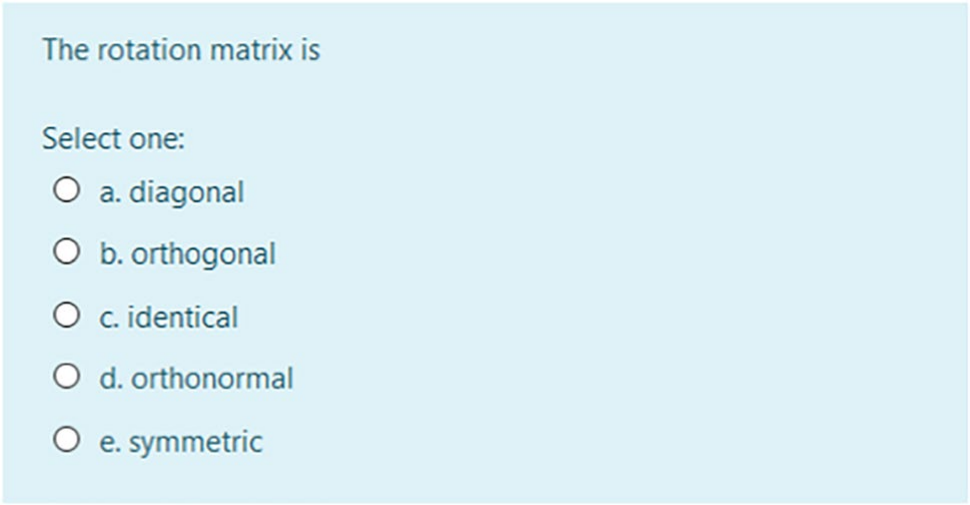
Multiple-choice question through Moodle quiz during the Photogrammetry and Remote Sensing course

An example of multiple choice question is represented in Fig. 4.

A maximum grade of 5 points was assigned to each quiz, all the questions having the same weight, equal to 1. The quiz tool gives the possibility to differentiate the questions weighting, based on the difficulty, type of question, or any arbitrary criterion; being the questions relative to fundamental concepts, the better choice seemed to give them the same weight and, consequently, the same maximum grade. Concerning the grade, the choice was to assign 1 point (maximum) to each correct answer and 0 points in case of incorrect answer. This is a non-punitive criterion, which helps to limit the students’ anxiety while performing the test. The passing grade was set to 3 points, so that the students should correctly answer to at least three questions to consider the quiz passed; otherwise, the quiz can be repeated for a maximum of three attempts. Each attempt was marked, and the final grade was given by the average of obtained marks. In such a way, the students are stimulated to be more concentrated and conscious while answering and they are encouraged to study before trying the quiz, so that the quiz is tackled with a positive attitude and a solid study base. This can help in making the quiz an educational activity, as it is in the teachers’ wish, rather than simply an assessment. To enhance this aspect, inside each quiz the questions were shuffled time by time for each attempt, to make the students more focused on the questions content rather than on the question sequence.

It should be noted that the obtained marks for the quizzes did not influence the final exam grade, which derived from the evaluation of the group presentation, the survey activity and data analysis carried out during the course, as well as some additional theoretical questions. In this way, the quizzes were intended to give a feedback firstly to the students, but also to the teacher: the students could verify their comprehension of the main concepts, have a self-assessment of their learning process, and clarify potential doubts, and the teacher could easily follow the learning process, eventually focusing and explaining again the topics where the students have given incorrect answers to the quizzes. On few occasions, the quizzes results gave to the students the opportunity to pose follow-up questions and to clarify some more difficult topics.

No time limit was imposed on the quizzes, so that they are freely accessible, without any predefined time limit (e.g., the quizzes can be set to be accessed in a specific time window or each question should be answered within a certain time limit). The only limit was imposed to the accessibility of the consecutive lessons: the quizzes were set in a way that the students should complete them and obtain a passing grade (at least 3 correct answers out of 5 questions) to have the access to the next unit material. This criterion gave a crucial role to passing the quiz; otherwise, the lesson material is unavailable, and it incited the students to keep up to date with the study and to tackle the quiz without anxiety but, on the contrary, with an increased self-confidence, given by the awareness of having studied. After attempting a quiz, a report is available to students, reporting the grade, the correct and incorrect answers, and eventual feedback on wrong answers, without revealing the correct answer, so that the students are motivated to review before attempting the quiz again. In case a quiz is attempted several times, multiple reports are available. The teacher report also includes the number of attempts per student with the corresponding grades and the number of quizzes answered by each student, to control their progress. The resulting reports can be exported from both teacher’s and students’ sides for a consequent teaching and learning assessment to understand the students’ performances.

The design and building of the quizzes were also the occasion to revise the courses educational objectives, to compile them in a proper way, and to link them with the quiz questions.

### Glossary

The glossary is a list of concepts built to be shared within the course. It is intended to be a tool to help the course participants to better focus their attention on the key concepts presented during the course and to make sure that they are clear.

Regarding the use of this tool within the GIS course, it consisted of two phases. In the first part of the course, in which the teacher provides the theoretical concepts for the correct management of geo-cartographic data, at the end of each lesson, the teacher asked the students to outline which important concepts they thought had been addressed during the lesson itself. Students, on a voluntary and free basis, entered the concepts in the glossary prepared on the Moodle platform dedicated to the course, but without giving a definition. At the end of the theoretical lessons, the teacher collected all the entries and removed duplicates. Then, students were asked to create groups of 2–4 people each, to make the learning outcomes potentially more significant (Swanson et al. 2019) following the team-based learning method. The teacher assigned (randomly) to each group some entries to be inserted in a final version of the course glossary, complete with their definitions. The definitions could be uploaded by individual students of the group but had to be shared with all group members. The group captain was responsible for checking that the group did its best work. At the end of the course, the teacher corrected the definitions inserted in the glossary, highlighting errors and inaccuracies in a constructive way. The corrected glossary was a solid basis for the students to prepare for the oral examination.

The glossary has proved to be both a useful tool for the student and a feedback for the teacher, who can check if students have understood the contents of the course. It can be also a valuable tool for the teacher to conduct the oral exam. In fact, the teacher does not evaluate the work of completing the glossary but takes note of the correctness and completeness of the descriptions entered by each group of students. During the exam, the teacher may ask for clarification on some descriptions entered and take into account the quality of the task.

In the Geomatics course, the compilation of a glossary was proposed to the students during the first lesson, asking them to enter some definitions according to their previous knowledge. It was compiled by 80% of the students. The compilation of a second glossary was proposed at the end of the course, to make the students aware of their own improvement. The low participation in the compilation of this glossary (less than 20%) compromised the autonomous verification of the students’ learning progress. Moreover, the students’ difficulties in approaching the proposed method did not allow to underline the main topics treated during the course.

### PBL—problem-based learning

The PBL is a didactic methodology whose goal is to resolve meaningful problems (Barrows 2000; Torp and Sage 2002; Hmelo-Silver 2004).

In the Geomatics field of knowledge, PBL teaching strategy is used as a common approach to teach Photogrammetry, Remote Sensing, and Laser Scanning at Aalborg University in Denmark (Höhle 2005). In Cartography and GIS disciplines, since the late 1990s, the most effective education has been carried out applying the PBL approach that offers a close integration between theory and practical training (Chen 1998). The advantages of this kind of training in the GIS field have been highlighted by several authors (Walsh 1992; Drennon 2005; Pawson et al. 2006).

PBL leads to deeper understanding and better competences compared to more traditional teaching methods, because students focus on attempting to resolve a realistic problem and learning skills that are later transferable to their careers.

Regarding the GIS courses at the Genoa University, the teacher usually applies such teaching technique as follows. For the students at the beginning of their GIS education, it could be better to use a version of the methodology called *problem-solving teaching*, in which the teacher guides the students through all the processes required to solve a problem, without leaving the scholars alone in finding the solutions (Hattie 2009). In fact, she guides some first practical exercises in the computer room to show and let the students experience how to import, visualize, and analyze the geo-cartographic data. Afterwards, the teacher poses some practical and operational problems to the students, asking to solve them. Usually, the solution is set up in classroom, through an initial discussion among the whole class. In the second part of the course, the teacher asks the students to work in small groups, with the help of a teaching support collaborator who is present in the classroom during the teamwork.

This methodology was adopted because of several studies showed its efficacy (Dochy et al. 2003; Hmelo-Silver et al. 2007), knowing that these effects can change depending on the students’ level of expertise (Kirschner et al. 2006; Michael 2006). Studies like the one conducted by Koh et al. (2008) found that PBL has a significant efficacy only when it is used with students that have already a noteworthy background of knowledge in the field in which the methodology is used. Moreover, coupling PBL with free and open-source software for geospatial (FOSS4G) science, as proposed during the GIS course in Genoa, is known to enrich the student experience (Mitasova et al. 2012; Ciolli et al. 2017), allowing them to explore different tools to solve real-world problems or even to develop customized tools.

Despite that, during the remote online lessons due to COVID-19, the problem-solving lessons were initially a monologue of the teacher in front of a monitor, without having any possibility to control what the students were able to do on their computers and without having any feedback from them on the encountered difficulties. Therefore, the teacher decided to adopt a different teaching strategy, abandoning PBL and experimenting TBL (Team Based Learning).

### TBL—team-based learning

The TBL is a group-based learning methodology that set the learning process in a collaborative way (Swanson et al. 2019). Several studies prove its efficacy (Fuchs et al. 1984; Phelps 2012).

The GIS students were involved in the practical exercises following these phases:

- Students were asked to form groups of 2–4 people each, to make the learning outcomes potentially more significant (Swanson et al. 2019). Even if the backgrounds of the students were heterogeneous, ranging from Civil and Environmental Engineering, Architectural Science to Geological Sciences, the resulting groups were generally homogeneous in origin.
- The teacher proposed to the groups some operative applications in which to analyze the territory or urban environments in GIS; each group had to choose two themes of interest.
- The teacher assigned to each group an operative exercise, choosing, if possible, between the two indicated by the group, trying to ensure that each exercise was assigned to two groups, possibly with different cultural backgrounds (Architects, Engineers, Geologists).
- Each operative exercise was prepared and led by the groups in charge. They researched the geographical data useful for the analysis, presented them to the rest of the class and proposed the analysis procedures. The two groups worked independently, therefore, during the presentation phase of the carried out preparatory work; the approaches and aims of the two groups were compared and enriched by the comparison and discussion.
- The teacher supported the two groups and the whole class in the application of the proposed elaborations.

By applying this teaching methodology, students were more involved in the analysis of the application of their interest, but also in the comparison and discussion with colleagues.

During the examination, the students are asked to discuss a teamwork, which can be either an in-depth analysis of the exercise prepared and guided in class, or an application of the same analysis procedures to a different geographical area, or, even better, an application that makes use of the analysis procedures seen in several operative exercises.

The TBL approach has been adopted also in the Geomatics, Geomatics for Monitoring, and Photogrammetry and Remote Sensing courses. The working groups have to process and analyze in a critical way the acquired dataset, consisting of:

- Spirit leveling, total station, GNSS measurements to frame in the official cartography, in the Geomatics course
- UAV photogrammetric imagery, Terrestrial Laser Scanner acquisitions and GNSS Ground Control Points (GCPs) coordinates, to monitor buildings or environments with different approaches in the Geomatics for Monitoring and in Photogrammetry and Remote Sensing courses

Finally, the students have to prepare a final presentation, simulating to interact with the customer in a transparent and critical way according to the obtained results.

### Didactic videos

As already stated, the introduction of the described novelties in the didactic process were planned in advance, regardless the COVID-19 health emergency outbreak. Nevertheless, the imposition of the remote and online attendance of the courses effectively conditioned in those subjects which require a field experimentation, as indeed Geomatics. This aspect was very challenging. The main objective was to provide the information in an alternative way, enough clear, captivating, and instructive to replace the fieldwork.

After discovering the potential effectiveness of the use of didactic videos (Ainsworth 1999; Ainsworth and VanLabeke 2004; van der Meij and de Jong 2006), the demonstrations about the use of the survey techniques instrumentation were fulfilled by means of video clips, recorded, and mounted by the teachers and commented during the lessons.

Three videos have been created, concerning the spirit leveling, the total station, and the GNSS survey techniques.

The video clips concern the instrument positioning, setting, and the point collimation. The shown phases include in detail both the preparation and the measurements, considering the main operative aspects to pay attention to during a survey campaign. A special emphasis is dedicated to the relationship between the final precision and the correct set up of the survey work.

The total duration of the three videos is about 15 min. Their goal is to give students the opportunity to have a preliminary approach to the survey instrumentation and its use. In the future, when it will be possible to attend courses and practical sessions again in presence, these videos will be used anyway to make students more aware of what they will do during the field demonstration.

### Indoor surveying with mobile devices

During academic year 2019/2020, it was not possible to go directly on the field for practical activities for all the Geomatics courses. The one that mainly suffered from this situation was Geomatics for Monitoring. For this course, the students are typically guided in an integrated surveying campaign involving several geomatics techniques, i.e., laser scanning, UAV, and terrestrial photogrammetry, GNSS, and topographic survey with total station, but it did not take place, due to COVID-19 lock-down. The data resulting from the survey campaign are usually processed, and the coherence of the different techniques is checked, e.g., the coordinates of GCPs surveyed with GNSS are compared with the ones surveyed by total station, and the laser scanner point cloud is compared with the one coming from photogrammetry. Moreover, the results of the present academic year are compared with the ones of the previous years, so to underline displacements, if any, as in a real-life monitoring. Despite the restrictions, the students were asked to perform the survey, staying at their houses and using their mobile phones to capture the images. The teacher and the teaching assistant proposed to use as object of the survey a readily available object in every home: flour that the students should model into a small heap surrounded by objects they can easily use as GCPs, so to scale the obtained model and to give it a reference frame. The students were instructed on how to take pictures and how to prepare the scene to perform the image shooting. They were also made aware that the photogrammetric software typically does not work properly in case of not textured objects, as the heap of flour is, so they were asked to add other ingredients to reach a good quality of the reconstructed 3D model. Each student worked independently and shared his/her results to the teacher, the teaching assistant, and the other students via videoconference during the lessons, preparing a short presentation to illustrate each step of the followed workflow, from the building of the heap to the placing and measuring of the GCPs, from the image capturing to the processing, together with a discussion on the several ingredients they have added and the effect on the photogrammetric 3D model. Once they all have obtained their own model with satisfying results, the students were asked to remove a small portion from the heap of flour and other ingredients, to perform the survey again, and to process the images so to obtain a different model of the same object to be compared with the original one and to discuss on the performed comparison.

This approach encouraged students’ individual work, creativity, and problem-solving skills but also involved them in a fruitful peer-to-peer comparison and discussion on the obtained results during the presentation of the carried-out work during the lessons. Also in this experience, the teacher and the teaching assistant acted as facilitators of the learning process, guiding the students through a trials and errors learning-by-doing strategy.

Finally, to overcome the lack of use of the other topographic instruments, the students were invited to experience them in laboratory and/or during other survey campaign of the following academic, and they were provided the didactic videos and other online references so to better understand how the main topographic instruments work.

## Teachers’ and students’ perception on the experimented innovative didactic tools

This section is dedicated to a critical analysis on the perception, from both students’ and teachers’ sides, on the innovative didactic tools that were experimented during the academic year 2019/2020 in Geomatics disciplines.

At the end of the GIS course, through the *Feedback* function on the Moodle platform, the teacher asked the students to express an opinion, anonymously and freely, on the instant poll, the glossary, and the TBL approaches. The interaction between the teacher and the students was considered good, but the impossibility of working in presence, without directly seeing and talking each other to cooperate and find solutions together, was considered “penalizing”. However, “it was interesting and useful to go and find out on first person the data on the various available geographical data portals.”

Despite these general difficulties, it emerges that the instant polls were considered “an appreciated and amusing incitement to study continuously; above all, it is useful to fix immediately the concepts tackled during the lesson, through a schematic and simple method.” Moreover, they were “a useful tool to verify the acquired knowledge, to understand if the tackled concept had been fully and correctly understood and learned.” Obviously, they were judged “useful if you study continuously, whereas they are less meaningful if you are not up to date with the study.” Finally, some students reported that “the instant polls made the lesson less dispersive.” These results are in line with other studies that analyzed the use of online polls in distance learning experiences (Christianson 2020; Shen et al. 2009, 2008; Baggaley et al. 2002): these activities make the didactic experience more active, both for students and teachers, and increase the level of interactivity.

Many students found the final glossary to be “a useful tool for studying, especially in view of the exam, as it helps to tidy up the information reported in the course slides, in order to extract the fundamental concepts.” Instead, the glossary activity at the end of each lesson was considered less useful, as the same words could be entered by several students. Considering this criticism, on the other hand, some students complained that “the number of instant polls and terms to be included in the glossary were too high” and “compiling the glossary required a huge effort and did not allow to distinguish the most important concepts from the rest of the provided information.” Despite the interactivity of the glossary, the students experienced a workload that made more difficult their learning process. Considering that these difficulties are not only related to their university experience, but also to the COVID-19 pandemic emergency (Mattarelli 2020), the teacher will use the instant poll instead of the glossary at the end of each theoretical lesson in the future academic years and will extract the most cited words to create the final glossary.

Following the current literature about the effectiveness of authentic online assessment (Lock and Redmond 2015; Marthur and Murray 2006), the TBL approach was considered “useful and structured in the correct way, because it allowed to interact and discuss the various operations that can be carried out.” It was therefore “a useful way to get a little more involved, although perhaps it would have been more engaging if the exercise lessons could be carried out in presence.” Asking groups with different backgrounds and coming from different university courses to perform the same operative exercises was an appreciated solution, for the possibility of analyzing the same topic from different points of view.

## Conclusions

During the academic year 2019/2020, an experimentation of innovative teaching techniques was conceived in Geomatics disciplines at the University of Genoa. It was planned before the COVID-19 emergency and then implemented adapting to the remote online teaching imposed by the disease outbreak. Several tools and techniques have been used to realize interactive lessons, promoting the student involvement: instant polls, quizzes, glossaries, didactic videos, and team- and problem-based learning (TBL, PBL) approaches. The theoretical basis and the practical method, with which these techniques have been implemented according to the specific needs and peculiarities of the course, have been here presented.

At the end of 1 year of experimentation, the assessment of the novelties introduced is widely positive. The approach based on TBL technique has been very formative for the students, who get more involved during the lessons. Instant polls, quizzes, and glossaries have made the teachers more aware about the learners’ feedback and allowed the students to verify the acquisition of the main concepts, to be better prepared for the final exams. Thus, the teachers’ proposal is to maintain these innovative teaching tools in the years to come, enriching them further to encourage the verification of the students’ own training, which is particularly important for their professional future.

Concluding, the positive reaction of the entire community, including teachers, students, and teaching support staff, to the innovations introduced in teaching and learning processes during the COVID-19 sanitary emergency should be particularly mentioned.

## Data Availability

Not applicable.

## Footnotes

1 The term “Third Mission” refers to all the activities of scientific, technological, and cultural transfer and productive transformation of knowledge, through which universities activate processes of interaction with the society, aiming to promote the economic and social growth, so that knowledge becomes a crucial instrument in obtaining economic, social, and cultural benefits and growth.

2 http://kids.unige.it/

3 http://festivaldelmare.unige.it/

4 https://pintofscience.it/

5 https://www.amiciacquario.ge.it/

6 http://www.istruzione.it/alternanza/

7 https://www.miur.gov.it/web/guest/-/linee-guida-dei-percorsi-per-le-competenze-trasversali-e-per-l-orientamento

8 https://www.isprs.org/education/benchmarks.aspx

9 https://www.wooclap.com/

10 The respondent has to briefly answer to a question.

11 The respondent answers to a question with few significant words; the result is represented as a cloud that enhances the more frequently written words, so that Word Cloud graphically represents keywords.

12 A list of questions/items is provided, along with a list of corresponding answers. The respondent must “match” the correct answers with each question.

13 The respondent chooses the correct answer within a given list of possible answers.

14 Similar to a short-answer question, with a numeric result.

15 The respondent has to order the given items, such as the cartographic product according to their scale.

16 https://moodle.org/

17 Missing words or phrases can be selected and added to text by dragging boxes to the correct location.

18 The respondent selects from two options (True or False) to give the answer.

19 Students select a missing word or a phrase from a dropdown menu to complete a sentence.
